# Social Inequality in Cigarette Consumption, Cigarette Dependence, and Intention to Quit among Norwegian Smokers

**DOI:** 10.1155/2015/835080

**Published:** 2015-07-26

**Authors:** Marianne Lund

**Affiliations:** Norwegian Institute for Alcohol and Drug Research, P.O. Box 565, Centrum, 0105 Oslo, Norway

## Abstract

*Background*. The study aim was to examine the influence of education and income on multiple measures of risk of smoking continuation. *Methods*. Three logistic regression models were run on cigarette consumption, dependence, and intention to quit based on nationally representative samples (2007–2012) of approximately 1 200 current smokers aged 30–66 years in Norway. *Results*. The relative risk ratio for current versus never smokers was RRR 5.37, 95% CI [4.26–6.77] among individuals with low educational level versus high and RRR 1.53, 95% CI [1.14–2.06] in the low-income group versus high (adjusted model). Low educational level was associated with high cigarette consumption, high cigarette dependence, and no intention to quit. The difference in predicted probability for having high cigarette consumption, high cigarette dependence, and no intention to quit were in the range of 10–20 percentage points between smokers with low versus those with high educational level. A significant difference between low- and high-income levels was observed for intention to quit. The effect of education on high consumption and dependence was mainly found in smokers with high income. *Conclusion*. Increased effort to combat social differences in smoking behaviour is needed. Implementation of smoking cessation programmes with high reach among low socioeconomic groups is recommended.

## 1. Introduction

While smoking rates among countries across the Western world are gradually decreasing, concerns over social inequality in smoking behaviour are increasing. Many studies have found an association between smoking behaviour and different measures of socioeconomic status (SES) such as education, income, and occupational class [1–3]. Smokers with low SES also have poorer cessation outcomes. This inequality pattern has been observed in studies of smoking cessation interventions and aggregated-level quit rates [4–6]. There is also some evidence of increasing social inequality in smoking behaviour and substantial health disparity consequences [7, 8].

In Norway, smoking rates are gradually declining, with a rate of 22% in 2014 in the adult population aged 16–74 years (13% are daily smokers). Norway has a strong welfare system and strives to be an egalitarian society that provides equal opportunities for all citizens. Despite reduced mortality in all educational groups, educational inequality in mortality increased in Norway in the period 1960–2000 [9]. Smoking is one mechanism behind this inequality [10]. Increased knowledge about social inequality in smoking behaviour can inform tobacco prevention efforts.

The pathways to successful quitting have been widely studied. Nicotine dependence is regarded as a primary barrier to giving up smoking and is predictive of smoking continuation [11]. Number of cigarettes per day (CPD) has often been used as a proxy for nicotine dependence, although some studies indicate that one should be cautious in interpreting high cigarette consumption as nicotine dependence. CPD is significantly associated with nicotine dependence, but differences in dependence are found to be independent of CPD level [12]. However, high cigarette consumption indicates a strong habit and illustrates aspects of dependence such as the time and effort the smoker dedicate to the behaviour [13].

Nicotine dependence has been widely measured in population-based surveys using different measurements like the Fagerström Test for Nicotine Dependence (FTND) and associated short versions such as the Heaviness of Smoking Index (HSI) and time to first cigarette in the morning (TTFC). The TTFC is likely the single item in the FTND that most strongly predicts addiction to nicotine, probably because morning smoking reflects the smoker's overnight withdrawal symptoms [14]. TTFC also shows good correlation with biological measures of nicotine ingestion [15].

The FTND, TTFC, and number of CPD are all predictive of smoking continuation and significantly associated with relapse following a quit attempt [16, 17]. Having an intention to quit smoking is strongly associated with quit attempts but is less consistent with quitting success [18].

Measures of nicotine dependence such as the FTND, HSI, and TTFC are significantly related to SES and show increasing dependence with decreasing SES [17, 19, 20]. The association between nicotine dependence and SES is also found in studies using biochemical measures of dependence, such as levels of cotinine in plasma [21].

However, the association between intention to quit and SES is less clear. Some studies report a positive relationship between low-SES smokers and intention to quit or quit attempts but reduced smoking cessation success among low-SES smokers [6, 22]. Other studies investigating the transtheoretical model of change report a higher proportion of smokers with a low educational level in the precontemplation stage (i.e., a smoker who does not intend to quit) [23, 24].

Norway is in the final stage of the tobacco epidemic, experiencing both a gradual decline in smoking prevalence and persistent inequality in smoking habits. In this situation, it is of interest to investigate differences in smoking behaviour that indicate smoking continuation. Risk of continued smoking is defined in three ways: high cigarette consumption, high cigarette dependence, and having no intention to quit. The aim of this study was to investigate the associations between education and income and risk of high cigarette consumption, cigarette dependence, and intention to quit. Because education and income are related, it was of interest to explore the combined effect of social inequality measures on risk of smoking continuation.

## 2. Method

### 2.1. Study Sample

Data were pooled from six cross-sectional datasets representative of the Norwegian population during 2007–2012. Approximately 1 200 respondents aged 16 years or older were surveyed by telephone during the autumn of each year by Statistics Norway. The study sample was 4 591 respondents aged 30–66 years. The lower age cut-off for inclusion was 30 years because younger adults may not have completed their education. A study sample aged 30 years or older also represents a population of individuals with an established smoking history, since more than half of daily smokers start smoking before age 18. Individuals who received early retirement pensions (*n* = 197) were excluded from the study sample, along with 89 individuals with missing education information. Survey response rates were 67% (2007), 57% (2008), 61% (2009), 54% (2010), 58% (2011), and 61% (2012).

### 2.2. Dependent Variables: Cigarette Consumption, Cigarette Dependence, and Absence of Intention to Quit

Three measures were used to capture risk of smoking continuation: cigarette consumption, nicotine dependence, and intention to quit. High* cigarette consumption *was defined as consumption of 15 CPD or more. Occasional smokers with an average weekly consumption above 105 were coded in the +15 CPD group.* Cigarette dependence* was the time to first cigarette in the morning (TTFC); individuals smoking within the first 30 minutes after awakening were defined as having high cigarette dependence and individuals who smoked 31 minutes or more after wakening had low cigarette dependence [14]. Although TTFC is most often referred to as a measure of* nicotine dependence*, it also captures nonpharmacological aspects of cigarette dependence such as psychosocial functions [13]. The term* cigarette dependence* is therefore preferred in the present study. Having no* intention to quit* was a measure of smokers' short- or long-term intention to quit; smokers with no intention to quit within the next 6 months and who also believed they would still be smoking in 5 years were defined as having no intention to quit.

### 2.3. Socioeconomic Measures: Education and Income

Two measures of SES were included as independent variables: educational level and income level. Educational level was recoded from the original nine-level variable to three levels: completion of lower secondary, upper secondary, and university levels. For the interaction analysis, we used a dichotomous measure of education with* high educational level *including completion of upper secondary school or university and* low educational level* representing completion of lower secondary school. Income was defined by combining the gross household income and marital status. Those with an annual household income above the median (NOK 700 000, ≈USD 160 000 or more) were coded in the high-income group. Medium income was NOK 300 000–699 000 (≈USD 36 000–50 000) and low income was below NOK 300 000. Those with a household income of NOK 300 000–699 000 and living alone were coded as having high income. In the study sample, 12% were in the low-income group (7% of the population sample, see Table 1). This is comparable to the percentage defined as having low income in Norway using the EU definition of 60% of median income [25].

### 2.4. Analyses

Data analyses were conducted in two parts. First, the representative sample was used to confirm socioeconomic differences in smoking status. For the multinomial regression, the smoking outcome category was defined as current and former smokers, with nonsmokers as the reference category. Results from this analysis are presented as a relative risk ratio (RRR) in Table 2. The characteristics of the population sample and study sample of all current smokers (daily and occasional smokers) are presented in Table 1. The logistic regression analysis included three binary outcomes reflecting risk of smoking continuation (cigarette consumption, cigarette dependence, and intention to quit), with education and income as independent variables. The models were adjusted for survey year, age, sex, and numbers of household members. Three logistic models were used to compute adjusted prediction (predicted probabilities) of the outcomes across the SES measures and marginal effects (differences in predicted probabilities) between different levels of SES (Table 3). Marginal effects show how the outcome changed for each change in the categorical independent variable. Marginal effects are estimated as average marginal effects, which means that other variables in the model are used* as observed* for each case. Tables 4 show the predicted probability for each combined group of education and income using the margins command (education # income) and the delta method was used to examine the statistical significance of group comparisons [26]. Only dichotomous measures of education and income were used for the combined effect (high versus low). All analyses were conducted using Stata statistical software (v.13).

## 3. Results

The proportion of individuals with low educational level (28.9%) was higher in the study sample of current smokers than in the population sample (17.3%) (Table 1). The proportion with low income level was 12.2% in the study sample and 7.2% in the population sample. One out of four current smokers reported having high cigarette consumption and no intention to quit, while one out of three reported having high cigarette dependence. Social inequality in smoking behaviour was confirmed. Educational differences were present in both the bivariate and the adjusted models, with RRR of 5.37, 95% confidence interval [4.26–6.77] for current compared with never smokers in the low educational level (Table 2). A significant association between income and current smoking was observed in the bivariate model. In the adjusted model, the RRR for current smoking was 1.53, 95% confidence interval [1.14–2.06] in the lowest compared with the highest income group.

Table 3 presents three separate logistic regression models for the outcome variables high cigarette consumption, high cigarette dependence, and having no intention to quit. Adjusted predicted probabilities for the outcomes of interest are presented for each SES group. Among current smokers, the probability of high cigarette consumption, high cigarette dependence, and having no intention to quit increased with reduced educational level (Table 3). The marginal effect shows a 15 percentage point increase between the highest and lowest educational groups in the predicted probability of having high cigarette consumption. The marginal effect of education on cigarette dependence showed a 19 percentage point increase.

Income produced somewhat different results than educational level, with the highest probability of the outcomes of high consumption and high cigarette dependence among those with medium income level. Low-income smokers had the same probability of being a high-consuming smoker as the high-income group, 26% and 25%, respectively (Table 3). The probability for cigarette dependence for high-, medium-, and low-income groups was 30%, 39%, and 37%, respectively.

Having no intention to quit was significantly associated with low educational level and low or medium income (Table 3). The adjusted predicted probability that a smoker with a low educational level would have no intention to quit was 31%, while the corresponding percentage for smokers with a high educational level was 19%.

Table 4 presents the adjusted predicted probabilities for the outcome variables for every combination of high and low educational levels and income. The education effect for the outcome cigarette consumption and cigarette dependence was only found among those with high income. There was a 10 percentage point difference in the probability of having a high cigarette consumption and being highly dependent on cigarettes between the highly educated with high income compared with those with a low educational level with high income (Table 4). A 10 percentage point difference was also found for cigarette dependence between those with high levels of both education and income compared with those with low levels of both education and income (“top-bottom” differences), but the difference did not reach statistical significance. An educational effect among the high-income smokers was also found for no intention to quit smoking, with a 9 percentage point difference. A significant “top-bottom” difference for having no intention to quit smoking was also observed, with a 19 percentage point difference in predicted probabilities. For example, a smoker with high educational level and high income had a predicted 21% chance of having no intention to quit smoking, while the corresponding number for a smoker with low educational level and low income was 40%.

## 4. Discussion

This study revealed a strong association between education and the outcomes indicating risk of smoking continuation: high cigarette consumption, high cigarette dependence, and having no intention to quit. Low income had an independent effect on intention to quit. The effect of education was only valid for those defined as having a high-income level. There was a 10–20 percentage point difference between high and low education level in relation to probability of high consumption, dependence, and no intention to quit.

Several studies confirm the importance of education for lack of smoking cessation and risk of smoking continuation [5, 19, 21, 27]. Possible explanations for the strong influence of education on smoking have included knowledge and cognitive resources, social networks, number of smokers and social norms regarding smoking in the social environment, health literacy, psychosocial stress, and health risk perceptions [28–30]. It has been suggested that education creates a culture that discourages smoking [31]. Being in a culture where smokers are in the minority and where norms against smoking dominate may make it easier for someone who smokes to quit. Stronger no-smoking norms among those with greater education may explain some of their lower risk of smoking continuation.

The strong association between education and smoking continuation may be ascribed to the association between delay discounting/impulsivity and education; several studies show that less educated individuals choose smaller, immediate rewards over larger, delayed rewards [32, 33]. This means that smoking would be valued more highly than future health. Current smokers discount delayed rewards more than never and former smokers and are more nicotine dependent than less dependent smokers, even when controlling for education [34, 35]. However, the association between education/income and nicotine dependence is stronger than the association between delay discounting and nicotine dependence [35]. A Norwegian study of adolescents found that both education and impulsivity predicted smoking initiation, but only education (not impulsivity) predicted smoking cessation. No interaction between education and impulsivity on smoking cessation was found [36].

The somewhat stronger relationship between education and smoking behaviour compared with income and smoking behaviour may vary by country [37]. Income had a curvilinear impact on high consumption. The high price of cigarettes in Norway may explain the low probability of high cigarette consumption in the low-income group, a finding in line with studies showing that low-SES groups are sensitive to increasing cigarette taxes [38]. However, this does not explain the low consumption levels among the high-income group in this study. Having low income may reduce cigarette consumption, but being financially deprived does not necessary imply an increased motivation to quit smoking.

Increases in the price of or tax on cigarettes are seen as having the most consistent positive impact, for example, the greatest potential to reduce inequality in smoking behaviour [38]. Interventions such as compulsory and national smoke-free policies and control on advertising, promotion and marketing of tobacco are regarded as having a positive or neutral impact; here, a neutral impact means that the effect would be equal regarding SES [38]. Norway scores relatively high on the cigarettes price score (20 out of 30 points) in the tobacco control scale in Europe [39]. Further tax increases are seen as problematic due to fear of increased cross-border trade with subsequent lost tax revenue and smuggling. Smoke-free legislation was introduced in Norway in 2004, with positive health effects among employees in the hospitality industry [40]. The impact of national smoke-free policies on reducing inequalities is found mainly in reduced social inequalities in passive smoking (nine out of 19 studies) [38]. Smoke-free legislation is expected to reduce the social acceptability of smoking, thereby contributing to the ongoing process of smoking denormalization. Whether denormalization processes have the same impact regardless of social status is unclear and highly debated (cf. the smoker stigma debate [41]).

Tobacco control interventions such as price/taxation increases and sales restrictions are considered highly effective because they affect most people. The population-level cessation support in Norway, with the exception of individual media campaigns that have been launched earlier, comprises a national quit line and a web site for smoking cessation support hosted by health authorities. Call rates to the quit line are higher among high-SES groups than low-SES groups and these SES differences are stable over time [42]. A study evaluating the Norwegian quit line is currently in progress. More intensive smoking cessation services implemented through the health care service with special focus on deprived areas have shown positive effects in reducing social inequality in smoking in England [43]. Reaching proportionally more low-SES smokers than high-SES smokers may compensate for the lower quit rates usually found in socially disadvantaged groups of smokers.

The present study results are consistent with others and show the need to increase motivation to quit and assist nicotine-dependent low-SES smokers to quit smoking. In addition, the present study has disentangled the effect of two SES measures (education and income) on three separate indicators of prolonged smoking. The results show substantial differences in motivation to quit between those with both high educational level and high income, compared with those with both low educational level and low income.

Many Western countries including Norway have made substantial progress in reducing smoking prevalence over the last two decades but have been unable to decrease social inequality in smoking behaviour. New population-based interventions are currently being debated, including plain packaging and harm reduction strategies such as use of electronic cigarettes. Given the high mortality rate from cigarette smoking and its contribution to health inequality, interventions that reduce smoking rates in low-SES populations are needed. However, few population-based interventions with an equity impact beyond those already identified, including price and tax increases, exist. A report from the Royal College of Physicians states that harm reduction strategies, such as electronic cigarettes, may have a potential role in preventing deaths from cigarette smoking and reducing social inequalities in smoking-related morbidity and mortality [44]. Further investigation on the potential role of electronic cigarettes to reduce social inequality in smoking is needed, both to assess their potential for helping nicotine-dependent smokers to quit as well as their potential to increase motivation to quit among smokers unwilling to quit smoking.

## Limitations

The cross-sectional design of this study makes it impossible to deduce causation. The validity of the outcome variables requires attention. Having high cigarette consumption, high cigarette dependence, and no intention to quit were used as indices of risk for smoking continuation. This is consistent with several studies reporting these measures in relation to unsuccessful cessation among hardcore smokers. In a longitudinal study, the predictive ability of high consumption, high dependence, and intention to quit was investigated in relation to continued smoking after 1 year. All components predicted smoking continuation, but nicotine dependence was the best predictor of smoking continuation [16].

## Figures and Tables

**Table 1:** Characteristics of the population and study samples (current smokers). Participants aged 30–66 years. Data were pooled from 2007 to 2012.

	Population sample (*N* = 4 600)	*n*	Study sample (current smokers, *n* = 1 282)	*n*
Age (mean, SD)	47.7 (10.2)	4 600	47.7 (9.7)	1 282
Male (%)	49.1	2 260	49.9	640
Educational level				
High	39.1	1 798	23.7	304
Medium	43.7	2 008	47.4	607
Low	17.3	794	28.9	371
Household income				
High	66.9	2 849	60.1	701
Medium	25.9	1 105	27.8	324
Low	7.2	308	12.2	142
Daily smokers (%)	20.4	937	73.1	937
Heavy smoking ≥15 CPD	7.9	365	28.5	365
TTFC ≤30 minutes	9.3	423	34.3	423
No intention to quit	7.1	327	25.7	237

**Table 2:** Adjusted multinomial regression for education and income according to smoking status with never smoker as reference group. Relative risk ratio (RRR) and 95% confidence interval. Bivariate and adjusted models.

	Model 1: bivariate relationship Never smoker = ref.		Model 2: adjusted for survey year, age, sex, and members of the household Never smoker = ref.	
	Current smoker	Former smoker	Current smoker	Former smoker
High education	Ref.	Ref.	Ref.	Ref.
Medium education	2.59 (2.21, 3.06)^***^	1.92 (1.63, 2.25)^***^	2.53 (2.12, 3.02)^***^	1.75 (1.47, 2.08)^***^
Low education	5.66 (4.61, 6.95)^***^	2.31 (1.84, 2.89)^***^	5.37 (4.26, 6.77)^***^	2.05 (1.59, 2.65)^***^
High income	Ref.	Ref.	Ref.	Ref.
Medium income	1.44 (1.22, 1.70)^***^	1.44 (1.22, 1.71)^***^	1.07 (0.89, 1.29)	1.16 (0.97, 1.40)
Low income	2.76 (2.12, 3.59)^***^	1.17 (0.84, 1.64)	1.53 (1.14, 2.06)^**^	0.84 (0.58, 1.21)

^***^ *P* < .001, ^**^ *P* < .01, ^*^ *P* < .05.

**Table 3:** Adjusted predicted probabilities and marginal effects (differences in predicted probabilities) of the outcomes high consumption, high cigarette dependence, and no intention to quit smoking by education and income. All variables included in each model, in addition to survey year, age, sex, and number of persons in household. Current smokers aged 30–66 years. Data were pooled from 2007 to 2012.

	High consumption		High cigarette dependence		No intention to quit	
	*N* = 1 147		*N* = 1 105		*N* = 1 142	
	Percent (95% CI)		Percent (95% CI)		Percent (95% CI)	
	Adjusted predicted probability	Marginal effects (difference in predicted probability)	Adjusted predicted probability	Marginal effects (difference in predicted probability)	Adjusted predicted probability	Marginal effects (difference in predicted probability)
Education						
High	17.9 (13.2, 22.6)	Reference	19.8 (14.7, 24.8)	Reference	19.1 (14.2, 24.1)	Reference
Medium	29.8 (26.1, 33.5)	11.9 (5.9, 18.0)^***^	36.3 (32.2, 40.3)	16.5 (10.0, 23.0)^***^	25.0 (21.4, 28.5)	5.9 (−0.2, 12.0)
Low	33.3 (28.1, 38.4)	15.4 (8.2, 22.5)^***^	39.0 (33.5, 44.4)	19.2 (11.6, 26.8)^***^	30.6 (25.6, 35.6)	11.5 (4.3, 18.7)^**^
Income						
High	25.1 (21.8, 28.4)	Reference	29.5 (25.9, 33.1)	Reference	20.8 (17.9, 24.3)	Reference
Medium	35.1 (29.8, 40.3)	10.1 (3.7, 16.3)^**^	39.3 (33.9, 44.7)	9.8 (3.2, 16.4)^**^	30.4 (24.8, 35.0)	9.6 (3.5, 15.7)^**^
Low	26.2 (19.0, 33.4)	1.1 (−7.0, 9.2)	36.6 (28.4, 44.8)	7.1 (−2.0, 16.2)	34.7 (25.1, 42.1)	13.9 (4.8, 23.1)^**^

^***^ *P* < .001, ^**^ *P* < .01, ^*^ *P* < .05.

**Table 4:** Margins (adjusted predicted probability) for high consumption of cigarettes, high cigarette dependence, and no intention to quit by education and income (margins income # education).

Education	Income	High cigarette consumption			*n*	High cigarette dependence			*n*	No intention to quit			*n*
		Margins	Unadjusted groups			Margins	Unadjusted groups			Margins	Unadjusted groups		
High	High	25.7	A		758	30.3	A		725	21.4	A		755
High	Low	26.7	A	B	76	34.2	A	B	74	31.8	A	B	76
Low	High	35.9		B	252	39.7		B	247	30.6		B	252
Low	Low	26.2	A	B	61	40.9	A	B	59	40.3		B	59

Margins sharing a letter in the group label are not significantly different at the 5% level.
